# Prospective nationwide audit of short-term outcomes after surgery for chronic pilonidal sinus disease in the Netherlands

**DOI:** 10.1007/s10151-025-03159-7

**Published:** 2025-06-11

**Authors:** E. A. Huurman, C. A. L. de Raaff, R. van den Berg, S. J. Baart, B. P. L. Wijnhoven, R. Schouten, E. J. B. Furnée, B. R. Toorenvliet, R. M. Smeenk

**Affiliations:** 1 Department of Surgical Oncology and Gastrointestinal Surgery, Erasmus MC Cancer Institute, Rotterdam, The Netherlands; 2 Department of Surgery, Albert Schweitzer Hospital, Dordrecht, The Netherlands; 3 Department of Biostatistics, Erasmus MC, Rotterdam, The Netherlands; 4 Department of Surgery, Flevoziekenhuis, Almere, The Netherlands; 5 Department of Surgery, University Medical Center Groningen, Groningen, The Netherlands; 6 Department of Surgery, Ikazia Hospital, Rotterdam, The Netherlands; 7 Dr. Zamenhofstraat 32A, 3061 Rotterdam, The Netherlands

**Keywords:** Pilonidal sinus disease, Successful wound healing, Minimally invasive techniques, Excision with secondary healing, Off-midline closure

## Abstract

**Background:**

The optimal surgical approach for chronic pilonidal sinus disease (PSD) remains unclear, resulting in variation in surgical practice. This study aimed to provide an overview of PSD subtypes and assess practice variation and short-term outcomes.

**Methods:**

A nationwide prospective observational cohort study was conducted. All patients with PSD and who underwent surgery were included during a 3-month inclusion period between March 1, 2020 and March 1, 2021. Primary endpoints were PSD classification and type and frequency of surgical approach. Secondary endpoints included symptoms, complications, recurrent open wounds, wound healing rate, time to wound healing, time to resume daily activities, reasons for selecting therapy, antibiotic prophylaxis, type of anesthesia, and hospital admission.

**Results:**

A total of 36 hospitals participated in the study, and 405 patients had chronic disease. The median follow-up period was 42 days. Mean age was 28 years and 335 (82.7%) patients were male. Simple (*n* = 213) and complex PSD (*n* = 192) was equally common. Twelve different treatment modalities were performed. Minimally invasive techniques were used the most (61.2%) and off-midline closure in only a small proportion of patients (5.7%). Minimally invasive techniques showed a significantly higher wound healing rate (41.1% vs 28.6%) and a shorter median time to closure (41 vs 78 days) compared to excision with secondary healing. They also had the shortest median time to resume daily activities (14 days).

**Conclusions:**

Simple and complex PSD were equally common. Practice variation in surgery is substantial. Minimally invasive techniques were most frequently performed and showed good short-term outcomes.

## Introduction

Pilonidal sinus disease (PSD) is an inflammatory condition affecting the sacrococcygeal region and can manifest as either an acute (abscess) or chronic condition. It occurs primarily in patients aged 14–40 years and described risk factors for disease development are male sex, an anatomically deep natal cleft and a positive family history [1–8]. Other factors that may influence disease severity and wound healing are obesity, smoking, presence of diabetes and the use of immunosuppressive medication [6, 7, 9–12].

There is broad consensus on the surgical treatment for acute PSD, that being incision and drainage of the abscess. However, the optimal surgical treatment algorithm for chronic PSD is unknown. In 2019, a survey from the Netherlands highlighted practice variation in the management of chronic PSD [13]. One reason for this is limited high-quality evidence to guide treatment decision-making and the absence of a universally accepted classification system guiding clinical practice [14]. Chronic PSD can present in different ways which may affect outcome and the choice for optimal surgical treatment. Furthermore, the time to complete wound healing and the risk of recurrence can vary according to surgical approach [15]. The objectives of this study were to provide an overview of the different PSD subtypes using a classification system and to assess the practice variation and short-term outcomes in the treatment of chronic PSD in the Netherlands.

## Materials and methods

The Strengthening of the Reporting of Observational Studies in Epidemiology (STROBE) statement was used in the design and implementation of the study and to prepare the manuscript [16]. The medical ethics committee of the University Medical Center Utrecht approved the study design (protocol number 20-671/C).

### Study design

All community hospitals and surgical clinics in the Netherlands that provide surgical care for patients with PSD were invited to participate in this prospective observational audit. All patients with PSD that were operated on at the participating hospitals were included during a 3-month inclusion period between March 1, 2020 and March 1, 2021. The inclusion period commenced at each participating hospital upon receiving study approval from the local ethics committee. Therefore, not all hospitals started simultaneously. The inclusion period could be extended by 1 month if elective surgical care capacity was scaled down in participating hospitals as a result of the COVID-19 pandemic. Patients who were already on the waiting list for elective PSD surgery were also contacted to participate in the study. All patients were treated according to the local hospital protocols.

### Data collection

In each participating hospital one surgeon and one surgical resident were responsible for the prospective data collection and for entering the anonymised data into a web-based database (Castor Electronic Data Capture, EDC^©^). After recruitment and signed informed consent, a patient electronic case report form (eCRF) was created in Castor EDC^©^. Preoperative and intraoperative data were processed after surgery and outcomes during follow-up visits at the outpatient clinic, scheduled for 1, 2 and 6 weeks post surgery (see supplementary file). Local hospital follow-up protocols were allowed because the aim of this study was to assess everyday practice. To standardize classification, the Dutch classification system was used (Table 1) [17]. Based on this classification, a distinction can be made between acute/chronic and simple/complex PSD. Acute PSD is identified as type II, and chronic PSD is identified as all other types. Simple PSD is identified as type I or type II, while complex sinus is identified as type III and type IV. Type V is not classified as either simple of complex but as a separate entity as this often requires a different treatment from the aforementioned types. For this study, only patients with chronic PSD were included for analysis.

**Table 1:** The Dutch classification system

Stage	Definition
Simple PSD	
Type Ia	One or more pits in the midline of the intergluteal cleft without symptoms
Type Ib	One or more pits in the midline of the intergluteal cleft with symptoms
Type II	Acute pilonidal abscess
Complex PSD	
Type III	Type 1b plus one or more sinus openings lateral to the intergluteal cleft. These sinus openings usually contain granulation tissue, blood, and/or pus. They are usually unilateral but can also present bilaterally
Recurrent pilonidal sinus	
Type IV	Recurrence of PSD after previous surgical treatment (excluding abscess drainage)
Chronic non-healing wound	
Type V	Chronic (usually hypergranulating) non-healing wound in the midline of the intergluteal cleft after previous surgery

*PSD* pilonidal sinus disease

### Eligibility criteria and outcome measures

This study included only patients aged 16 years or older with symptomatic PSD who underwent surgery and provided written informed consent. Primary endpoints included classification of PSD subtypes, types and frequency of surgical treatment. Secondary endpoints included the incidence and type of symptoms, the occurrence and type of postoperative complications, recurrent open wounds, the rate of successful wound healing (defined as complete wound closure with no residual discharge at the last outpatient visit), the median time to achieve successful healing in days, the median time to resume daily activities in days, reasons for selected therapy by the surgeon, the use of antibiotic prophylaxis, the type of anesthesia, and the type of hospital admission.

### Statistical analysis

Statistical analysis was performed using SPSS version 24.0 or higher (SPSS Inc., Chicago, IL, USA). Continuous variables were evaluated for normal distribution using visual inspection. For continuous variables, normally distributed data were reported as means ± SD, and medians with interquartile range were used in skewed data. Categorical data were reported in frequencies and percentages. Associations with categorical variables and type of treatment were analysed with the chi-squared test. The time until wound healing and resumption of daily activities was visualized using the Kaplan–Meier method and analysed with the log-rank test.

## Results

**Fig. 1:**
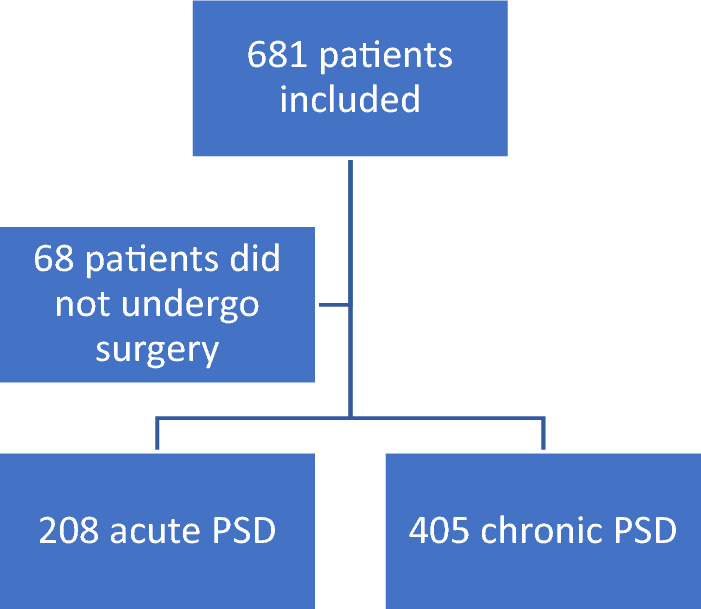
Study population

A total of 36 (out of 66) hospitals participated in the study and 681 patients were included. However, 68 patients did not undergo surgery for various reasons including resolved symptoms and waiting list issues caused by the COVID-19 pandemic. This resulted in a cohort of 405 patients with chronic PSD and 208 with acute PSD (Fig. 1). The median follow-up period after surgery was 42 days (IQR 29–49).

### Patient characteristics

The mean age (SD) of the patients was 28 (10) years and 335 (82.7%) of included patients were male. A total of 197 (48.6%) patients had previously undergone at least one operation for PSD (Table 2).

**Table 2:** Patient characteristics (*n* = 405)

	*N* = 405
Mean age (SD)	28 (10.01)
Male (%)	335 (82.7)
Mean BMI (SD)	26 (4.4)
Missing,* n*	13
Prior surgical treatment of PSD (%)	197 (48.6)
History of PSD abscess (%)	116 (28.6)
Diabetes mellitus (%)	8 (1.9)
Immunosuppressant use (%)	8 (2.0)
Smoking (%)	129 (32.3)
Missing,* n*	5
Family history of PSD (%)	73 (18.8)
Missing,* n*	16
Sedentary occupation (%)	177 (45)
Missing,* n*	12

### Preoperative symptoms

Simple (type I) and complex PSD (type III or IV) were equally common in our study population, and symptom severity tended to increase with PSD complexity (Table 3).

**Table 3:** Symptoms according to the classification system

	Simple		Complex		
Type, *N* = 405	Ia (*n* = 22)	Ib (*n* = 191)	III (*n* = 97)	IV (*n* = 73)	V (*n* = 22)
Itch (%)	11/22 (50)	90/185 (48.6)	53/96 (55.2)	43/71 (60.6)	11/22 (50)
Loss of wound fluid (%)	13/22 (59.1)	139/187 (74.3)	85/96 (88.5)	59/73 (80.8)	20/22 (90.9)
Loss of blood (%)	6/22 (27.3)	100/188 (53.2)	69/96 (71.9)	44/73 (60.3)	18/22 (81.8)
Loss of pus (%)	9/22 (40.9)	104/188 (55.3)	71/96 (74)	49/73 (67.1)	17/22 (77.3)
Embarrassment (%)	11/22 (50)	96/182 (52.7)	56/95 (58.9)	46/69 (66.7)	18/22 (81.8)

### Surgical treatment

**Fig. 2:**
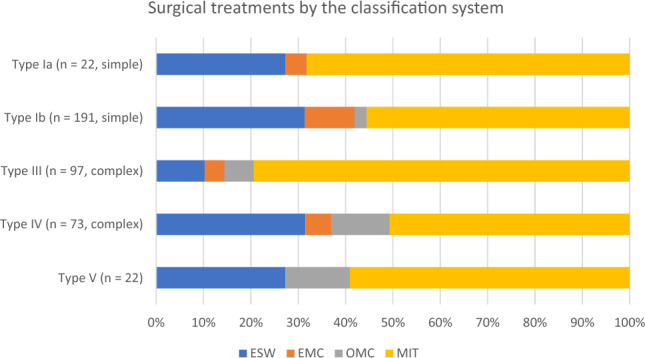
Surgical treatments according to the classification system. *ESW* excision with secondary wound healing, *MIT* minimally invasive techniques, *OMC* excision with off-midline closure, *EMC* excision with midline closure

**Fig. 3:**
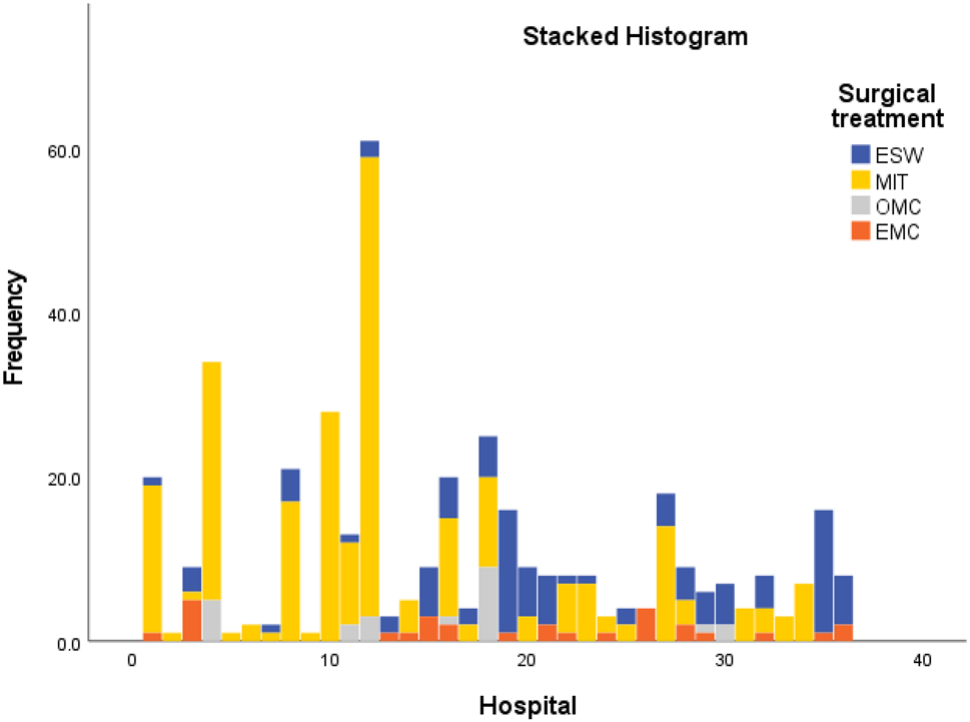
Surgical treatments by hospital. *ESW* excision with secondary wound healing, *MIT* minimally invasive techniques, *OMC* excision with off-midline closure, *EMC* excision with midline closure

Excision with secondary wound healing (ESW) was performed in 105 (25.9%) patients. Excision with midline closure (EMC) was performed in 29 (7.2%) patients. Minimally invasive techniques (MIT) were the most frequently performed, accounting for 248 (61.2%) patients. Excision with off-midline closure (OMC) was less common and was performed in only in 23 (5.7%) patients (Table 4). MIT were performed for both simple and complex PSD. Off-midline closure was mainly utilised for complex PSD (Fig. 2). The number of patients registered per hospital per year, as well as the type of surgical treatment, are presented in Fig. 3.

**Table 4:** Surgical treatments

	*N* = 405
ESW (%)	105 (25.9)
Without VAC therapy	104 (25.7)
With VAC therapy	1 (0.2)
EMC (%)	29 (7.2)
OMC (%)	23 (5.7)
BCL	20 (4.9)
Karydakis	3 (0.7)
MIT, *n* (%)	248 (61.2)
Pit picking	26 (6.4)
Pit picking + phenol	61 (15.1)
Pit picking + laser	114 (28.1)
Pit picking + silver nitrate	2 (0.5)
Pit picking + hydrogen peroxide	18 (4.4)
Deroofing	11 (2.7)
Bascom 1	12 (3.0)
Debridement	4 (1.0)

*ESW* excision with secondary wound healing, *VAC* vacuum assisted closure, *EMC* excision with midline closure, *OMC* excision with off-midline closure, *BCL* Bascom cleft lift, *MIT* minimally invasive techniques

### Perioperative data

MIT were most frequently performed under local anesthesia (47.6%) and conducted in an outpatient setting (44.8%). MIT were employed on the basis of the surgeon’s experience and hospital protocol (Table 5). The reported reasons for surgeons performing ESW and EMC were that they learned these techniques during their residency. The reason for OMC was determined by the surgeon’s experience with this procedure and current evidence.

**Table 5:** Perioperative data

	ESW (*n* = 105)	EMC (*n* = 29)	OMC (*n* = 23)	MIT (*n* = 248)
Antibiotic prophylaxis (%)	2/105 (1.9)	4/29 (13.8)	17/23 (73.9)	3/247 (1.2)
Anesthetic type (%)				
Local	9/105 (8.6)	3/29 (10.3)	1/23 (4.3)	117/246 (47.6)
Locoregional	24/105 (22.9)	6/29 (20.7)	4/23 (17.4)	38/246 (15.4)
General	72/105 (68.6)	20/29 (69)	19/23 (82.6)	92/246 (37.4)
+ Local	12/105 (11.4)	4/29 (13.8)	11/23 (47.8)	58/246 (23.6)
Type of hospital admission (%)				
Daycare	93 (88.6)	24 (82.8)	22 (95.7)	135 (54.4)
Short stay	2 (1.9)	2 (6.9)	1 (4.3)	2 (0.8)
Outpatient clinic	10 (9.5)	3 (10.3)	0 (0)	111 (44.8)
Reason for selected therapy				
Learned during residency	46/104 (44.2)	13/27 (48.1)	1/23 (4.3)	19/245 (7.8)
Hospital protocol	29/104 (27.9)	3/27 (11.1)	4/23 (17.4)	101/245 (41.2)
Based on own experience	26/104 (25)	11/27 (40.7)	12/23 (52.2)	106/245 (43.3)
Based on current evidence	3/104 (2.9)	0/27 (0)	6/23 (26.1)	19/245 (7.8)

*ESW* excision with secondary wound healing, *EMC* excision with midline closure, *MIT* minimally invasive techniques, *OMC* excision with off-midline closure

### Short-term outcomes

At 6 weeks postoperatively, patients who underwent MIT exhibited the lowest percentage of persisting symptoms compared to the other groups (Table 6). The group undergoing EMC had the highest complication rate at 34.5%, whereas the MIT group had the lowest complication rate at 10.4%. Most complications were wound infections and abscess formation. In the MIT group, 11 patients experienced a recurrent open wound during the follow-up. Five of them were scheduled for reoperation.

**Table 6:** Short-term outcomes

	ESW (*n* = 105)	EMC (*n* = 29)	OMC (*n* = 23)	MIT (*n* = 248)
Complication (%)	12/105 (11.4)	10/29 (34.5)	4/23 (17.9)	26/248 (10.5)
Seroma	1 (1)	–	1 (4.3)	4 (1.6)
Bleeding	5 (4.8)	1 (1)	–	2 (0.8)
Abscess formation	2 (1.9)	4 (13.8)	1 (4.3)	7 (2.8)
Infection	1 (1)	5 (17.2)	2 (8.7)	7 (2.8)
Skin necrosis	–	–	–	1 (0.4)
Skin burn	–	–	–	1 (0.4)
Persistent open wound	1 (1)	–	–	2 (0.8)
Hypergranulation	1 (1)	–	–	1 (0.4)
Type unknown	1 (1)	–	–	1 (0.4)
Recurrent open wound (%)	–	–	–	11 (4.4)
Wound dehiscence (%)	NA	17/26 (65.4)	14/22 (63.3)	NA
Wound dehiscence size (length) (%)	NA			NA
≤ 2 cm		11/17 (64.7)		
2–5 cm		3/17 (17.6)	9/14 (64.3)	
> 5 cm			3/14 (21.4)	
Unknown		3/17 (17.7)	2/14 (14.3)	
Skin closed after dehiscence at last outpatient visit (%)	NA	5/15 (33.3)	5/12 (41.7)	NA
Symptoms (%)				
Week 1	66/81 (81.5)	16/22 (72.7)	15/20 (75)	126/170 (74.1)
Week 2	59/82 (72)	15/21 (71.4)	15/19 (78.9)	119/171 (69.6)
Week 6	44/83 (53)	13/23 (56.5)	12/23 (56.5)	67/175 (38.3)

*ESW* excision with secondary wound healing, *EMC* excision with midline closure, *MIT* minimally invasive techniques, *OMC* excision with off-midline closure, *NA* not applicable

### Primary wound healing

In the group with EMC, 17/26 patients (65.4%) experienced wound dehiscence. For those in the OMC group, 14/22 patients (63.3%) had wound dehiscence. In the majority of cases, the wound dehiscence was less than 2 cm in length. After 6 weeks, 13/22 patients (59%) in the OMC group exhibited closed wounds, whereas 14/26 patients (54%) in the EMC group had closed wounds.

### Secondary wound healing

**Fig. 4:**
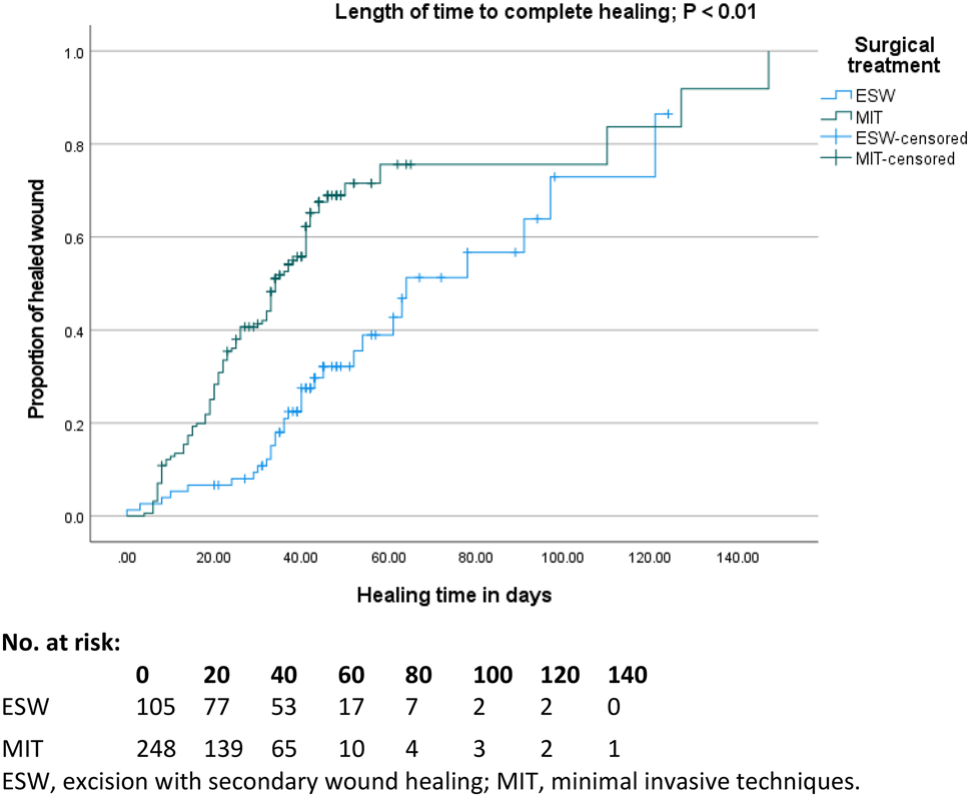
Kaplan–Meier curve demonstrating the length of time to heal and number at risk table. *ESW* excision with secondary wound healing, *MIT* minimally invasive techniques

Of the ESW operations performed, 30/105 (28.6%) resulted in successful healing after the median follow-up of 42 days. In the MIT group 102/248 (41.1%) achieved successful wound healing, which was statistically higher compared to the ESW group (*P* = 0.026). Median time to closure was 78 days (95% CI 49–106) for ESW, compared to 41 days (95% CI 36–46) for MIT (Fig. 4, *P* < 0.01).

### Resumption of daily activities

**Fig. 5:**
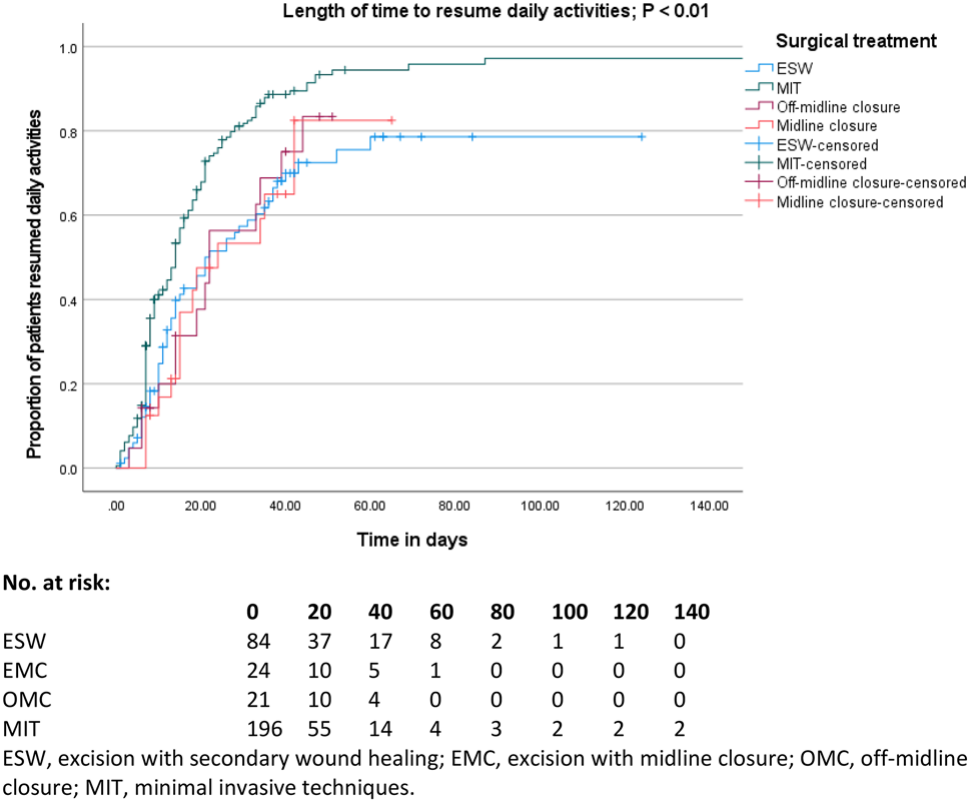
Kaplan–Meier curve demonstrating the length of time to resume daily activities and number at risk table. *ESW* excision with secondary wound healing, *EMC* excision with midline closure, *OMC* off-midline closure, *MIT* minimally invasive techniques

After a median follow-up of 42 days, 54/84 (64.3%) of patients resumed their daily activities to their previous level after ESW, 15/24 (62.5%) after EMC, 166/196 (84.7%) after MIT, and 14/21 (66.7) after OMC (*P* < 0.01). Median time to resume daily activities was 21 days (95% CI 10–31) for ESW, 24 days (95% CI 3–45) for EMC, 14 days (95% CI 12–16) for MIT and 22 days (95% CI 20–24) for OMC (Fig. 5, *P* < 0.01).

## Discussion and conclusion

This prospective nationwide snapshot study revealed that simple and complex PSD were equally common in most Dutch hospitals. It also showed that there is variation in surgical treatment of PSD, with MIT as the most frequently performed procedure in the participating hospitals. Surgical treatment was also performed in patients without symptoms (type Ia). This suggests that there may be insufficient consideration given to disease severity when consenting from patients for surgical treatment. This was also demonstrated in a 2019 survey in the Netherlands, which revealed that only 1.8% of the surgeons (in training) used a classification system for PSD [13] and 21% treated asymptomatic patients surgically. A Dutch guideline has since been developed and this was published in 2022 to provide surgeons with a more evidence-based guidance in treatment choice [17]. This guideline, however, was not yet published before the initiation of this snapshot study, and this study also shows that many Dutch surgeons did not apply existing evidence and guidance on PSD treatment in their daily practice. 

The choice of a particular technique seems to be determined by the surgeon’s preference, skill set and hospital protocol more than by the stage of the disease, as technique was not related to complexity of PSD. Following MIT, the most frequent performed procedures were ESW and EMC. These techniques, chosen primarily because they were taught during residency, were performed slightly more for simple PSD. However, they are not recommended according to current guidelines, with EMC being discouraged [17–20]. The least common treatment was OMC, mainly used for complex PSD and only by a few surgeons that have some experience with the technique.

When reviewing the short-term outcomes of different treatment modalities, the complication rate seemed relatively low after MIT and high following EMC. This makes sense as primary closure carries a higher risk of complications/infections whereas the small wounds in MIT are left open in most cases after the procedure. The percentage of wound dehiscence was high after both EMC and OMC. However, the majority of the dehiscences were small in size in both groups and from our experience will eventually heal.

In this study, MIT demonstrated favourable short-term outcomes. A significantly higher rate of successful wound healing and a shorter median time to closure were seen compared to ESW (41.1% vs 28.6%; 41 vs 78 days). The median time to resume daily activities after MIT was only 14 days. This duration is longer than reported in the literature [21], possibly as a result of the use of the outpatient visit date instead of the actual date.

A recent publication by Brown et al. includes a survey on the treatment preferences of UK surgeons in managing PSD [22]. Just as in the Netherlands, two-thirds of practitioners implemented interventions not recommended by guidelines, such as ESW as well as EMC. There was a tendency to prefer invasive excision procedures over minimally invasive ones, even when the latter might be suitable. Surveys from other countries have also indicated persistent use of these techniques [23–25]. In the present study, ESW and EMC were still applied in 33.1% of cases. This indicates the need to educate surgeons as the current literature suggests that ESW and EMC are not supported by the best available evidence and guidance [17, 26, 27]. Recovery from these procedures is too long and failure rates unacceptably high [15]. According to the study by Brown et al., it appears that patients in the UK also tend to reject this treatment, likely because of the need for prolonged nursing care [28].

The literature highlights more favourable alternatives, including MIT and flap surgery. In our study, we observed good outcomes with MIT in the short term, consistent with the literature. Although these methods seem to offer advantages such as decreased pain, accelerated wound healing, and quicker resumption of daily activities owing to reduced surgical trauma, it is essential to acknowledge a trade-off with higher recurrence rates [15, 21, 29]. In our study, 11 recurrent open wounds were already observed during the short follow-up period. Both short-term benefits and a higher risk of recurrence should be discussed when obtaining informed consent from patients for MIT.

Among alternative techniques, asymmetrical closure approaches, such as Karydakis and Bascom’s cleft closure, have gained popularity, with an increasing number of surgeons preferring this method compared to a decade ago [25]. Flap surgeries appear to offer advantages in terms of wound healing duration and recurrence compared to ESW [15, 30–34]. The present study shows that flap surgeries are only conducted to a limited extent in the Netherlands. A likely explanation for their restricted use is the insufficient training provided during residency and the extended learning curve associated with these procedures compared to EWS or EMC. The percentage of wound dehiscences after flap surgeries is higher than those reported in the literature [32, 35, 36]. Reasons for this could include differences in patient populations, modification of the operative technique, or that the surgeons performing the procedure have not completed the learning curve. No definitive conclusions could be drawn on the basis of the results in this study because of the relatively small number of patients.

The strength of this snapshot study is that all consecutive patients undergoing PSD surgical treatment were included prospectively without any selection bias based on severity of disease, age or comorbidity. Level of missing data was very low. However, this study has also its limitations. Firstly, non-teaching hospitals were represented less in this cohort because of the lack of surgical residents in these hospitals. Many patients with PSD are probably also treated in these hospitals, and the physicians working there may have different treatment strategies. It is therefore uncertain whether the results of this study are completely representative of Dutch practice (with approximately 70 hospitals). Secondly, one hospital included a high number of MIT (*N* = 56) because of hospital protocol, and this might have implications on the final results presented in this study. Thirdly, the variability in case load and department volume across the participating hospitals, as well as the potential impact of surgeon experience, may influence the outcomes. Finally, the study’s short follow-up time is a disadvantage as important data, especially with regard to wound healing and recurrence, might have been missed. This could, however, not be prevented as this study aimed to focus on everyday practice in PSD, which seems to include only a short follow-up in the Netherlands.

In conclusion, this prospective nationwide snapshot study confirms that simple and complex PSD were equally common in our study population. Practice variation in PSD surgery is substantial in the Netherlands and is irrespective of disease severity. MIT was most frequently performed in the participating hospitals and showed good short-term outcomes. Future prospective studies are necessary to explore the long-term outcomes of this cohort. Additionally, education is required as some techniques that are not in line with guideline recommendations are still being performed frequently, whereas those that are recommended are hardly performed at all.

## Data Availability

No datasets were generated or analysed during the current study.
